# Exploring Prognostic Biomarkers of Acute Myeloid Leukemia to Determine Its Most Effective Drugs from the FDA-Approved List through Molecular Docking and Dynamic Simulation

**DOI:** 10.1155/2023/1946703

**Published:** 2023-06-15

**Authors:** Md. Murshid Alom, Md Omar Faruqe, Md. Khademul Islam Molla, Md Motiur Rahman

**Affiliations:** ^1^Laboratory of Molecular Health Science, Department of Genetic Engineering and Biotechnology, University of Rajshahi, Rajshahi 6205, Bangladesh; ^2^Department of Computer Science and Engineering, University of Rajshahi, Rajshahi 6205, Bangladesh

## Abstract

Acute myeloid leukemia (AML) is a blood cancer caused by the abnormal proliferation and differentiation of hematopoietic stem cells in the bone marrow. The actual genetic markers and molecular mechanisms of AML prognosis are unclear till today. This study used bioinformatics approaches for identifying hub genes and pathways associated with AML development to uncover potential molecular mechanisms. The expression profiles of RNA-Seq datasets, GSE68925 and GSE183817, were retrieved from the Gene Expression Omnibus (GEO) database. These two datasets were analyzed by GREIN to obtain differentially expressed genes (DEGs), which were used for performing the Gene Ontology (GO), Kyoto Encyclopedia of Genes and Genomes (KEGG) pathway, protein-protein interaction (PPI), and survival analysis. The molecular docking and dynamic simulation were performed to identify the most effective drug/s for AML from the drug list approved by the Food and Drug Administration (FDA). By integrating the two datasets, 238 DEGs were identified as likely to be affected by AML progression. GO enrichment analyses exhibited that the upregulated genes were mainly associated with inflammatory response (BP) and extracellular region (CC). The downregulated DEGs were involved in the T-cell receptor signalling pathway (BP), an integral component of the lumenal side of the endoplasmic reticulum membrane (CC) and peptide antigen binding (MF). The pathway enrichment analysis showed that the upregulated DEGs were mainly associated with the T-cell receptor signalling pathway. Among the top 15 hub genes, the expression levels of *ALDH1A1* and *CFD* were associated with the prognosis of AML. Four FDA-approved drugs were selected, and a top-ranked drug was identified for each biomarker through molecular docking studies. The top-ranked drugs were further confirmed by molecular dynamic simulation that revealed their binding stability and confirmed their stable performance. Therefore, the drug compounds, enasidenib and gilteritinib, can be recommended as the most effective drugs against the ALDH1A1 and CFD proteins, respectively.

## 1. Introduction

Acute myeloid leukemia (AML) is a hematopoietic malignancy (cancer) in which too many immature blood-forming cells are accumulated in the bone marrow that also interferes with the production of normal blood cells, such as red blood cells, platelets, white blood cells, and other components [1]. It is the most common type of leukemia in adults, accounting for roughly 80% of all cases [2]. It is distinguished by the clonal expansion of immature “blast cells” in the peripheral blood and bone marrow, which results in ineffective erythropoiesis and bone marrow failure. Chemotherapy, monoclonal antibody therapy, stem cell transplant, and CAR-T cell therapy are designed for the treatment of AML [3]. Chemotherapy works as a remissive for AML patients; however, sometimes, within 12 months of the treatment, the cancer returns, for which the physician recommends the stem cell transplant, which is unfavorable for health and also costly. Immunotherapy-based methods have been proven to be appealing for AML patients, who have become resistant to chemotherapy over the previous decade. This innovative therapy can target antigens on the leukemic stem and blast cells, resulting in decreased toxicity [3]. The clinical suspects have been diagnosed as AML patients when they have at least 20% of bone marrow blast cells [4]. Flow cytometry-based immunophenotyping is used for diagnosing and determining the lineage of leukemic cells [5, 6]. The majority of AML patients display clonal heterogeneity at the time of diagnosis, showing the presence of both a founding clone and at least one subclone [7]. During a patient's relapse, different patterns of dynamic clonal evolution occur, which most likely contribute to therapeutic resistance [8]. Once AML was considered incurable, 35% to 40% of 60-year-old patients or younger patients are now cured, and patients older than 60 years are cured by 5% to 15% [6]. Advances in AML treatment for younger patients gained significant improvement, while the same for elderly patients remains unclear [9]. Even with the treatments so far developed, up to 70% of patients aged 65 years and over die from their disease within a year of diagnosis [10]. In this circumstance, finding the potential key biomarkers and therapeutic targets of AML would immensely promote the medication and reduce casualty. The study of high throughput sequencing can provide an understanding of the pathology and molecular mechanism of AML. GEO database is a worldwide web-based depository for high-throughput gene expression, where various functional genomic data sets are archived and freely distributed [11]. The identification of appropriate compounds against the prognostic biomarkers is a very crucial requirement nowadays. A prognostic biomarker is a genetic indicator that predicts the likelihood of a future clinical event, disease recurrence, or disease progression in a known population. The biological characteristics of the biomarker are objectively measured and evaluated for predicting the course of a disease or a response to a therapeutic intervention among the patients. Combined computational and experimental methodologies have been proven to be beneficial for identifying and developing novel promising molecules. This study was performed using two datasets from the GEO database and obtained the results by exploring the molecular function of mRNA related to AML. It has been demonstrated that the molecular dynamics simulation (RMSD, hydrogen bond, Rg, SASA, RMSF, and MM-PBSA) indicates the degree of movements and conformational changes within the interaction sites of protein-ligand, which supports our understanding on how ligands interact and associate with proteins [12–14].

A detailed understanding of the molecular mechanism for AML pathogenesis is likely to provide a rationale for developing and designing an appropriate therapy. FDA approved a list of drugs for medicating AML, but doctors speculate about prescribing the most effective drug from the list. However, it is a bit difficult for a doctor to select a drug having the most effectiveness. So, the doctors are to prescribe a drug for a patient as if they experiment on the patient. Our study is aimed at exploring the prognostic biomarkers and the molecular pathways related to AML and thereby identifying the best-matched specific therapeutic compounds for the appropriate treatment of AML through molecular docking and molecular dynamic (MD) simulation.

## 2. Materials and Methods

**Figure 1:**
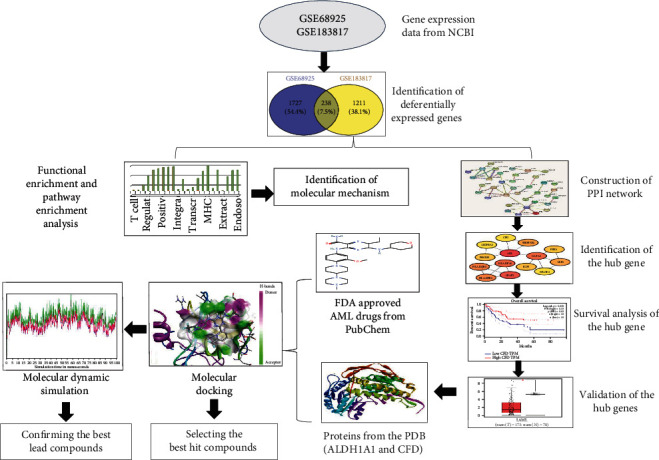
Schematic diagram outlining the workflow of our proposed approach.

The workflow of the present study is presented in Figure 1.

### 2.1. RNA-Seq Data

This study used the keyword “Acute Myeloid Leukemia” and searched on the NCBI's GEO database (https://www.ncbi.nlm.nih.gov/geo/) and selected two datasets, GSE68925 [15] and GSE183817 [16], because they were associated with AML prognosis. For this study, seven data samples were selected from 10 samples having accession number GSE68925, of which four (GSM1686542, GSM1686543, GSM1686544, and GSM1686545) were leukemic blast cells as case samples, and three (GSM1686546, GSM1686547, and GSM1686548) were healthy hematopoietic stem/progenitor cells (CD34+ HSPC) considered as control samples. The remaining three (GSM1686539, GSM1686540, and GSM1686541) were excluded because of nontranscriptomic samples. Another gene expression profile dataset with accession number GSE183817 has 13 peripheral blood samples, of which four (GSM5571742, GSM5571743, GSM5571744, and GSM5571745) were chosen as *de novo* AML patients, and three (GSM5571752, GSM5571753, and GSM5571754) were healthy samples taken as control. The remaining samples were excluded as they are refractory secondary AML (GSM5571746, GSM5571747, and GSM5571748) and refractory/relapsed AML (GSM5571749, GSM5571750, and GSM5571751). The selected datasets were analyzed from *Homo sapiens*, and the tissue system and cell type were also relevant to AML for both datasets. Detailed information about the data sets is shown in Table 1.

### 2.2. Identification of Differentially Expressed Genes in AML

GREIN (GEO RNA-seq Experiments Interactive Navigator) [17] is an interactive online analysis tool using an R-based automated pipeline GREP2. RNA-seq raw sequencing data from the GEO database were simultaneously downloaded and processed through GREIN. The absolute log fold change (log (FC) > 1) and *p* value < 0.05 were considered as the selection criteria of differentially expressed genes (DEGs) for AML samples from two RNA-seq datasets. The distribution of the DEGs in the datasets was presented as a Venn diagram using the Venny online tool [18]. Volcano plots and heat maps were generated for each dataset through SRplot (http://www.bioinformatics.com.cn/srplot) [19].

### 2.3. GO and Pathway Enrichment Analysis of DEGs

The Database for Annotation, Visualization, and Integrated Discovery (DAVID, https://david.ncifcrf.gov/) [20] is an online tool that dispenses a comprehensive set of functional annotation tools to recognize biological information behind a large list of genes. Gene Ontology (GO) and Kyoto Encyclopedia of Genes and Genomes (KEGG) annotations were analyzed using this database. During analysis, we uploaded an integrated gene list and selected the official gene symbol at the select identifier option as well as choose the gene list at the list type setting to perform the GO and KEGG analysis. The *p* value < 0.05 was defined as significant enrichment.

### 2.4. Constructions of PPI Network and Identification of Hub Genes

The STRING (https://string-db.org) database was used to build the protein-protein interaction (PPI) network by setting up their interaction score > 0.4 [21], and Cytoscape software (version 3.9.0, [22]) was used to visualize and analyze the PPI network model. The cytoHubba plugin ranks nodes in a network and identifies the hub genes based on their network properties. Based on the ranking method of maximal clique centrality (MCC), this study defined the top 15 genes as the hub genes.

### 2.5. Survival Analysis of Hub Genes

Gene Expression Profiling Interactive Analysis (GEPIA, http://gepia2.cancer-pku.cn/) is an interactive bioinformatics online tool used for investigating the RNA-sequencing data from the TCGA and the GTEx projects [23]. Using GEPIA, the relationship between key gene expression and AML prognosis can be elucidated following log rank. For generating survival plots of the DEGs, the GEPIA web tool was used with the parameters of methods (overall survival), group cutoff (high 50% and low 50%), hazard ratio (yes), 95% confidence interval (yes), and axis units (months) with the dataset of AML. The difference between the cutoff high 50% and low 50% was considered statistically significant when the *p* value was < 0.05. The possible effects of hub genes were assessed using the overall survival (OS) study [24]. These hub genes were designated as biomarkers because of their role in the prognosis of AML.

### 2.6. Validation of Hub Genes

GEPIA (http://gepia2.cancer-pku.cn/) includes different functions such as tumor and normal differential expression analysis, profiling of cancer types or pathological stages, and survival analysis of patients. GEPIA was used to exhibit the box plot for revealing the results to validate and analyze the expression of key genes. Log (FC) > 1 and *p* value < 0.05 were set to screen the data's validity.

### 2.7. Molecular Docking

The three-dimensional structures of the complexes were predicted based on the binding properties of each ligand with their cognate proteins. The prognostic biomarkers ((ALDH1A1 (PDB ID: 7JWW [25]) and CFD (PDB ID: 5NAT [26])) were taken as model proteins after validation of the hub genes docked with each of the FDA-approved drugs for AML treatment. The three-dimensional structures of the prognostic proteins and FDA-approved drugs were retrieved from the protein data bank (PDB) database and PubChem database, respectively.

The protein structures were preprocessed by PyMOL software (version 2.5) [27], where water and other nonessential residues were removed from the proteins. The potential energy of the proteins was minimized using Swiss-PDB Viewer software [28] for better optimization. Hydrogen atoms have been added to proteins to make them protonated for better docking performance. Molecular docking was carried out by PyRx software (version 0.8) [29], which is commonly used for docking studies.

The grid box was generated, and for ALDH1A1, the center points of the box were *X* = 44.018, *Y* = −14.84, and *Z* = 19.702; and dimensions are *X* = 76.3418565941, *Y* = 66.6992212296, and *Z* = 55.7972505379 (all are in Angstrom). For CFD, the center points of the box were *X* = −1.4345, *Y* = −0.0011, and *Z* = 13.0908; and dimensions are *X* = 43.7299689674, *Y* = 43.2379607773, and *Z* = 55.4447107124 (all are in Angstrom). Molecular visualization is important in modelling analysis after docking the selected compounds with the target proteins. The docking results were visualized and analyzed using BIOVIA Discovery studio client 2021 [30].

### 2.8. Molecular Dynamic Simulation

Molecular dynamic simulation studies greatly improve our understanding of protein stability when it binds to a ligand. The ligand and target protein are physically separated, and the ligand is then permitted to bond into the groove of the target after “specified durations of moves” in its conformational space. Internal (torsional angle rotations) or external (rotational angle rotations) changes the structure of the ligand involved in the motion (rotations and translations). It is also more realistic to evaluate the molecular recognition between the ligand and the target protein. Due to the significant energy dissipation for each conformation, this technique takes a longer time to determine the best-docked conformer. Fast optimization methods and grid-based tools have largely transformed this flaw in recent years, making simulation more user-friendly [31]. The molecular dynamic simulation was done in YASARA dynamics [32] using the AMBER14 force field [33]. The cubic simulation cell was built, complexes were tuned, and hydrogen bond networks were oriented. The steepest gradient techniques were employed using a simulated annealing method to minimize the protein complexes using a TIP3P water solvation model (0.997 g/L1, 25 c, 1 atm) [34]. The simulated system was neutralized at 0.9% NaCl, 310 K, and pH 7.4 [35]. The electrostatic interaction was calculated using the particle mesh Ewald method, with a radius of 8 Å cutoff. The simulation cell was stretched to 20 Å on both sides of the system so that the protein could move freely. A Berendsen thermostat was employed to maintain the simulation temperature constant [36]. The simulation was run at 1.25 frames per second, with the trajectories saved every 100 ps. It was carried out for over 100 ns, and subsequent trajectory analyses were implemented by SciDAVis software available at http://scidavis.sourceforge.net. All snapshots were then subjected to YASARA software's MM-Poisson–Boltzmann surface area (MM-PBSA) binding free energy calculation using Formula (1) below [37].

(1) $$\begin{array}{c} \text{BFE}=\text{EpotR}+\text{EsolR}+\text{EpotL}+\text{EsolL}-\text{EpotC}-\text{EsolC}\cdots \cdots \cdots \cdots \cdots \cdots . \end{array}$$

Here, BFE: binding free energy; EpotR: EpotReceptor; EsolR: EsolvReceptor; EpotL: EpotLigand; EsolL: EsolvLigand; EpotC: EpotComplex; EsolC: EsolvComplex.

With AMBER 14 as the force field, the MM-PBSA binding energy was estimated in this case using built-in YASARA macros, where bigger negative energies indicate better binding [38].

## 3. Results

### 3.1. Identification of DEGs

**Figure 2:**
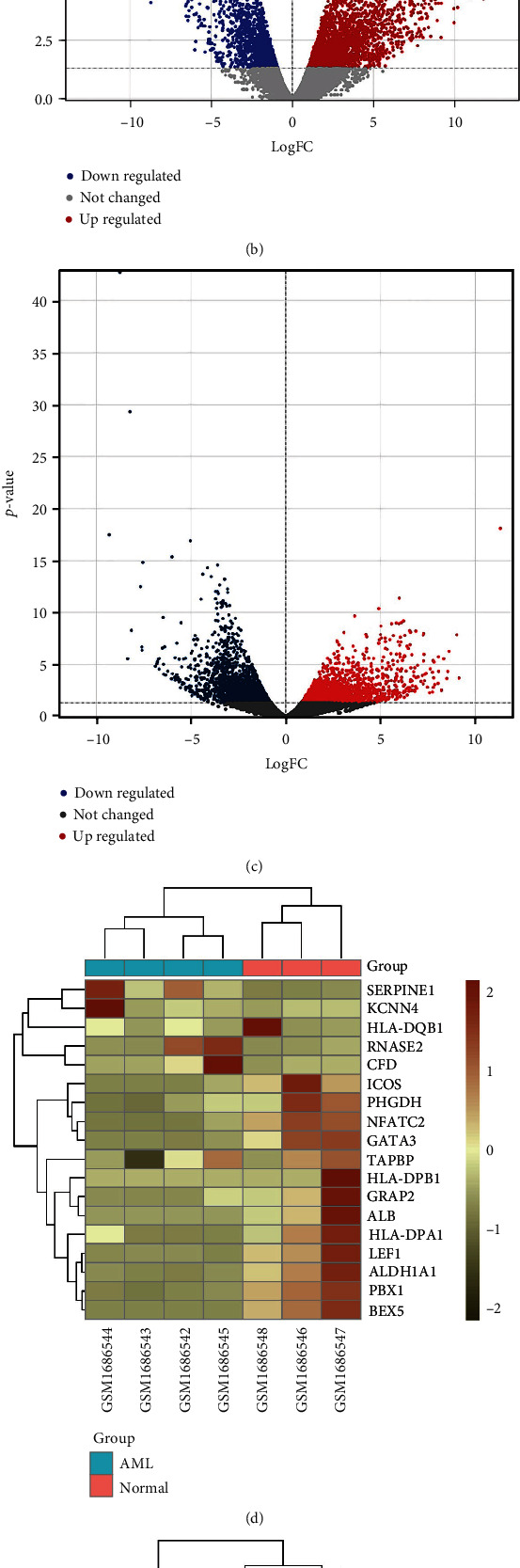
Identification and confirmation of DEGs. (a) Venn diagram showing the integrated data from the two GEO datasets. (b, c) Volcano plots of the two datasets, GSE68925 and GSE183817; here, red dots: upregulated DEGs, blue dots: downregulated DEGs, and black dots: genes with no significant difference in expression. (d, e) Heatmap for top 20 integrated DEGs of each dataset (GSE68925 and GSE183817); here, brownish: relatively upregulated DEGs and greenish: relatively downregulated DEGs.

GSE68925 and GSE183817 were investigated to identify DEGs by comparing blood samples among healthy and AML patients. A total of 1965 differential genes in the GSE68925 dataset (1221 upregulated and 744 downregulated genes) and 1449 differential genes in the GSE183817 dataset (614 upregulated and 835 downregulated genes) were identified by using GREIN (Table 1). The upregulated and downregulated genes were detected according to the logFC (fold change in log2 scale (usually)). Figures 2(b) and 2(c) show the volcano plots of the two datasets.

### 3.2. Integration of DEGs

The aim of the integration of DEGs is to find out the common DEGs from two datasets. The two datasets were integrated using the online tool Venny 2.1.0 [18], which identified 238 integrated DEGs (Table 2, Figure 2(a)). We took 95 integrated DEGs (including 38 upregulated and 57 downregulated genes) for our analysis based on logFC value. The heatmap of the top 20 DEGs in each dataset is shown in Figures 2(d) and 2(e).

### 3.3. GO Enrichment Analysis

**Figure 3:**
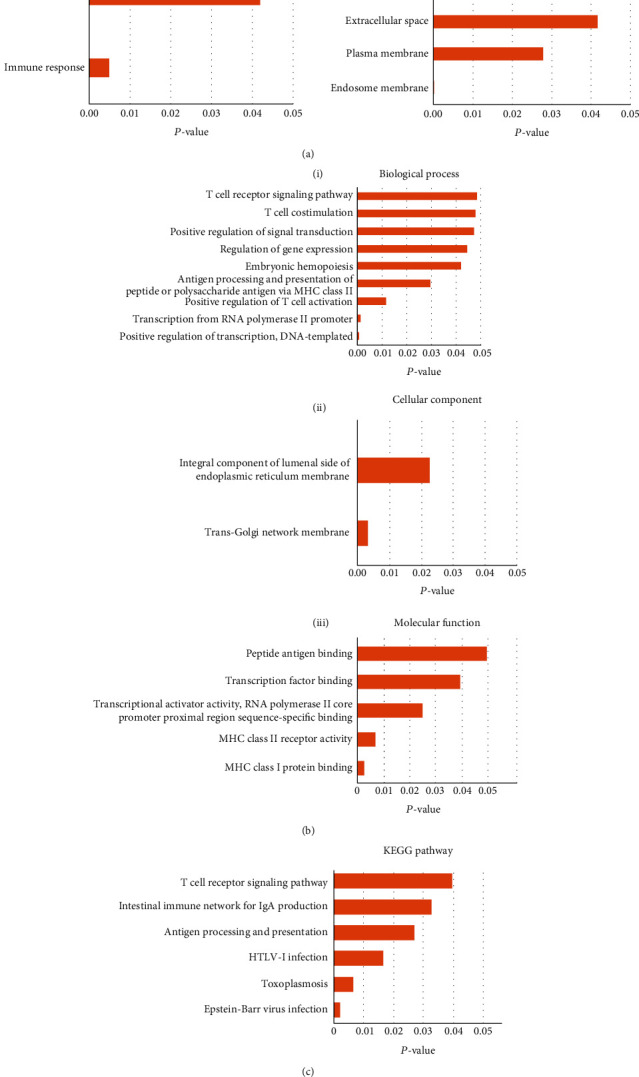
GO enrichment and pathway enrichment of DEGs. (a) Significant GO enrichment of the upregulated DEGs: (i) biological process and (ii) cellular component. (b) Significant GO enrichment of the downregulated DEGs: (i) biological process, (ii) cellular component, and (iii) molecular functions. (c) Significant pathway enrichment of downregulated DEGs.

GO is a technique for locating classes of genes or proteins that are overrepresented in a large collection of genes or proteins and may be related to disease characteristics. DEGs are categorized according to their biological process (BP), cellular component (CC), and molecular function (MF) in GO analysis. The functional processes of the DEGs were performed using GO analysis, shown in Tables 3(a) and 3(b) and Figures 3(a) and 3(b).

In BP, the upregulated DEGs were enriched in the inflammatory response, immune response, CC, extracellular region, extracellular space, plasma membrane, endosome membrane, and MF serine-type endopeptidase activity. In BP, the downregulated DEGs were enriched in the T cell receptor signalling pathway and costimulation, regulation of gene expression, embryonic hematopoiesis, antigen processing and presentation of peptide or polysaccharide antigen via MHC class II, positive regulation of T cell activation, transcription from RNA polymerase II promoter, positive regulation of transcription, and DNA templated. While in CC, the downregulated DEGs were enriched in integral components of the lumenal side of the endoplasmic reticulum membrane, trans-Golgi network membrane, and in MF, peptide antigen binding, transcription factor binding, transcriptional activator activity, RNA polymerase II core promoter proximal region sequence-specific binding, MHC class II receptor activity, and MHC class I protein binding.

### 3.4. Pathway Enrichment Analysis

Pathway enrichment analysis is frequently used to understand high throughput molecular data and produce hypotheses about the underlying biological processes of studies. The downregulated DEGs were associated with the T cell receptor signalling pathway, the intestinal immune network for IgA production, antigen processing and presentation, HTLV-I infection, toxoplasmosis, and the Epstein-Barr virus infection, according to functional enrichment analysis of integrated DEGs (Table 3(c) and Figure 3(c)).

### 3.5. PPI Network Analysis of DEGs

**Figure 4:**
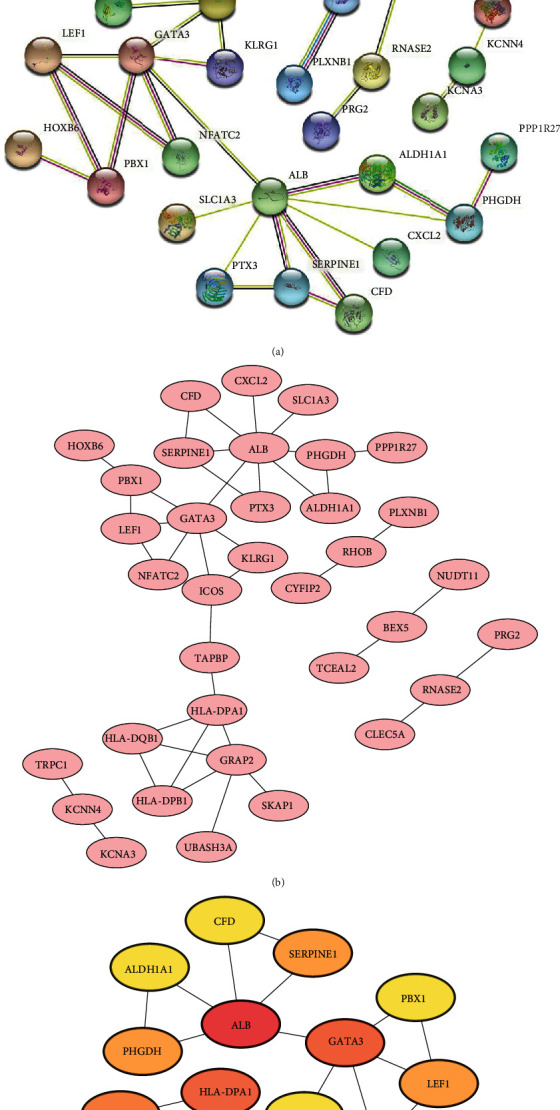
PPI network analysis of DEGs. (a) PPI network of the integrated DEGs. NB: circles indicate genes, and lines represent the PPI. Each protein structure is shown in the corresponding circle. (b) PPI analysis of integrated DEGs based on Cytoscape. (c) Interconnection of identified hub genes.

PPI network analysis methods are an effective way to quicken our understanding of the biochemical and molecular interactions that underlie pathogenesis. A PPI network was built using the STRING online database (Figure 4(a)), which was also used to analyze the integrated DEGs obtained by screening. The interaction score was set to medium confidence 0.4, which resulted in 91 nodes and 40 edges. By the MCC topological analysis method, the cytoHubba plugin [39] of Cytoscape [22] was used to screen out the top 15 hub genes (*ALB*, *GRAP2*, *HLA-DPA1*, *GATA3*, *HLA-DQB1*, *HLA-DPB1*, *SERPINE1*, *LEF1*, *PBX1*, *PHGDH*, *ICOS*, *KCNN4*, *ALDH1A1*, *NFATC2*, and *CFD*) as shown in Figures 4(b) and 4(c). Finally, the module's hub genes with strong connections were discovered. These genes play an important role in the features and progression of the disease.

### 3.6. Survival Analysis of Hub Genes

**Figure 5:**
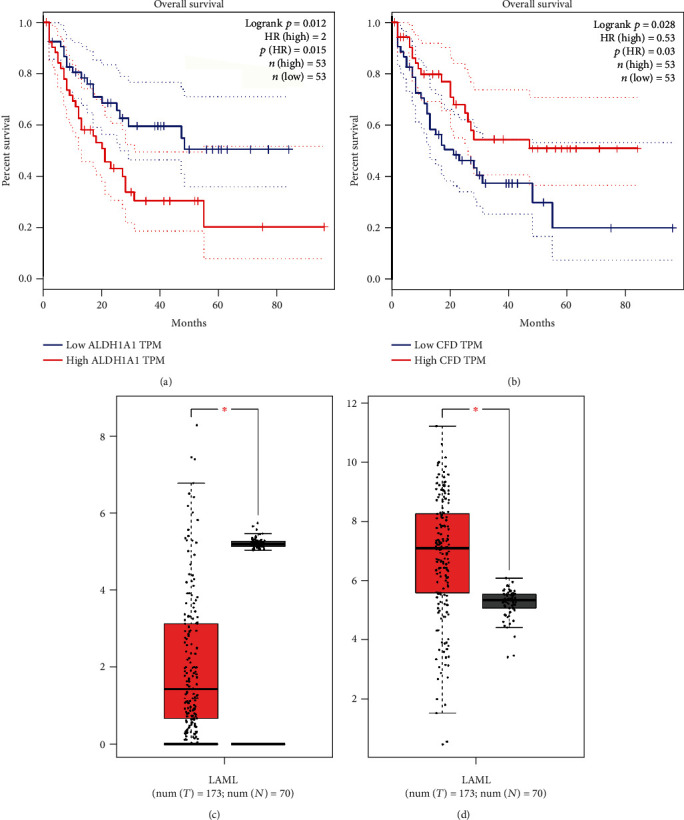
Survival analysis and validation of the hub genes in AML. Survival analysis of ALDH1A1 (a) and CFD (b). Here, the blue dotted lines indicate the ranges of expression levels of low ALDH1A1 TPM and low CFD TPM, and the red dotted lines indicate the ranges of expression levels of high ALDH1A1 TPM and high CFD TPM. The verification of mRNA expression of ALDH1A1 (c) and CFD (d) in terms of the boxplots.

Survival analysis is a vital aspect of medical statistics and is widely used to develop prognostic indices for mortality, recurrence, and investigation of therapeutic outcomes. In the GEPIA database, the Kaplan–Meier survival analysis [40] was performed on individuals with AML. Based on the gene median expression, 106 AML patients were split into two equal groups: high and low gene expression. According to the curves shown in Figures 5(a) and 5(b), we found that the OS was lower in the high ALDH1A1 expression group than that in the low expression group (*p* = 0.012). Besides, the OS was lower in the low CFD expression group than that in the high expression group (*p* = 0.028). The other hub genes were not shown to be significantly linked to AML prognosis due to *p* > 0.05 (Figure S. 1). According to the analysis, two biomarkers related to AML prognosis, ALDH1A1 and CFD, were identified.

### 3.7. Validation of Hub Genes

Data validation is a method that confirms the final data by matching it to a set of standard characteristics data. GEPIA was used to verify the essential genes' dependability. According to the database, genes were deferentially expressed in normal and AML samples. The expression level of ALDH1A1 and CFD was significantly higher in the tumor group compared with the normal group. In both cases, the expression level was found to be 173 for tumor and 73 for normal condition. These findings offer fundamental information about the expression pattern of the major key genes and their impacts on the survival of AML patients for further research. The outcomes were shown as boxplots (Figures 5(c) and 5(d)). After evaluating the primary data, we discovered that the results were compatible with our analysis and found ALDH1A1 and CFD genes.

### 3.8. Molecular Docking

The molecular docking analysis is used to simulate the interaction of atomic level between a small molecule and a protein, allowing us to characterize how small molecules behave at the binding site of target proteins and enabling a better understanding of biological processes. Docking is a method for finding a suitable ligand that fits into the sites of a protein, energetically and geometrically. In other words, it is the study of how two or more molecules, such as ligands and proteins, interact with each other. The ligands can drive the functional changes of the target molecules, which are determined by their binding in the active sites of the targets [20, 21]. Based on all the analyses mentioned earlier, *ALDH1A1* and *CFD* have been identified as novel therapeutic targets for AML. The crystal structures of the two biomarkers were retrieved from the Protein Data Bank (PDB), which are PDB ID: 7JWW [25] for ALDH1A1 and PDB ID: 5NAT [26] for CFD. The FDA-approved drugs for AML treatment were targeted and analyzed to achieve a docking score for determining the most effective drug for each of the biomarkers.

Docking studies were carried out to investigate the molecular binding pattern of the compounds within the active pocket of protein surfaces. Tables 4 and 5 show the results of the interaction between the two proteins and the compounds. By analyzing the docking interactions for ALDH1A1 protein, we found that enasidenib exhibited the lowest binding affinity (best binding score) of −10.8 kcal/mol, followed by −10.7, −10.5, and −10.1 kcal/mol with prednisone, daunorubicin, and doxorubicin, respectively. In the case of CFD protein, the gilteritinib exhibited the lowest binding affinity (best binding score) of −8.3 kcal/mol, followed by −7.7, −7.3, and −7.2 kcal/mol with glasdegib, enasidenib, and cerubidine, respectively.

**Figure 6:**
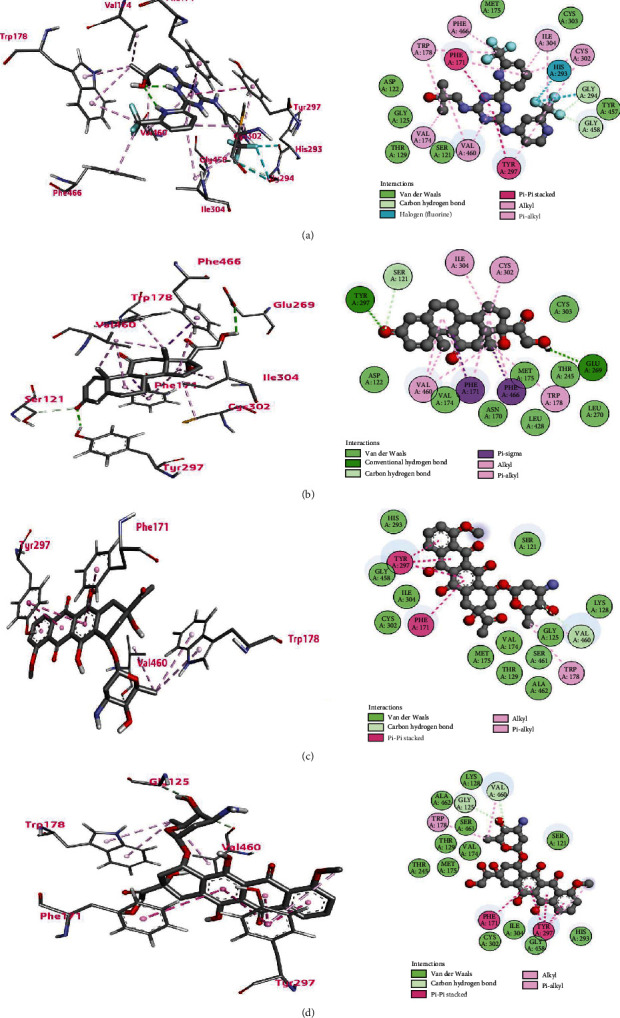
Ligand binding with ALDH1A1 protein for the top four compounds: (a) enasidenib, (b) prednisone, (c) daunorubicin, and (d) doxorubicin. Here, the left panel represents the 3D view of the binding site of the complex, and the right panel represents the 2D view of the protein-ligand interaction. The H-bonds are shown by dashed green lines (darker color), while the other dashed lines represent hydrophobic interactions and other types of intermolecular interaction.

**Figure 7:**
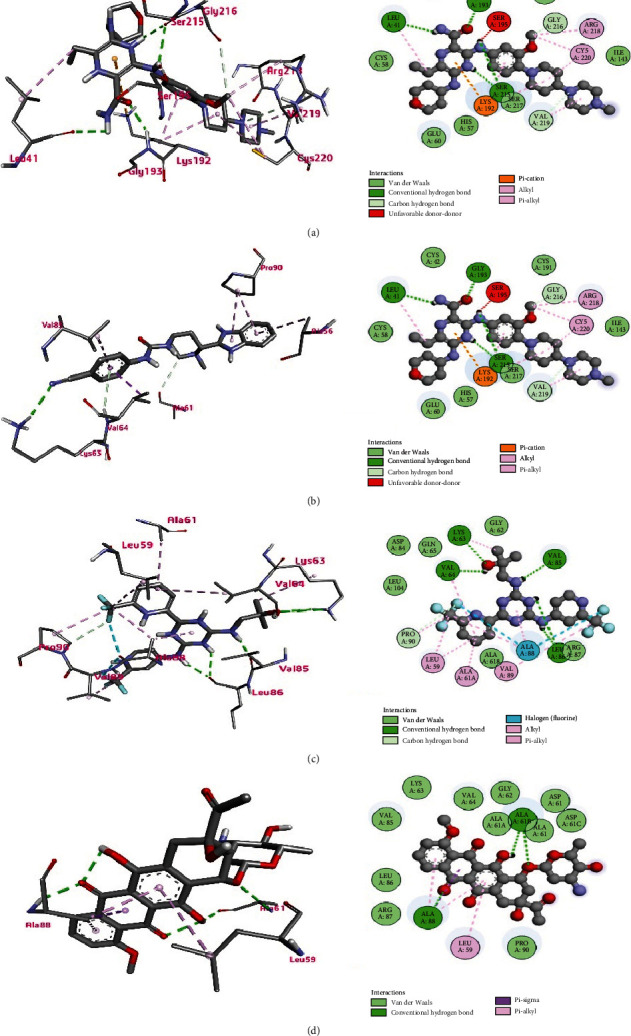
Ligand binding with CFD protein for the top four compounds: (a) gilteritinib, (b) glasdegib, (c) enasidenib, and (d) cerubidine. Here, the left panel represents the 3D view of the binding site of the complex, and the right panel represents the 2D view of the protein-ligand interaction. The H-bonds are shown by dashed green lines (darker color), while the other dashed lines represent hydrophobic interactions and other types of intermolecular interaction.

In the interaction of ALDH1A1, the enasidenib formed carbon-hydrogen bonds with GLY458 and GLY294 residues (bond distances in Table 6); the van der Waals bonds with ASP122, GLY125, THR129, SER121, TYR457, CYS303, and MET175; a halogen bond with HIS293; alkyl bonds with TRP178, PHE466, VAL174, VAL460, CYS302, and ILE304; and pi-pi stacked bonds with PHE171 and TYR297 (Figure 6). In the case of CFD, gilteritinib formed conventional hydrogen bonds with SER215, GLY193, and LEU41 residues; carbon-hydrogen bonds with VAL219, GLY216, and SER217, (bond distances in Table 6); the van der Waals bonds with CYS58, GLU60, HIS57, ILE143, CYS191, and CYS42; a pi-cation bond with LYS192; and alkyl bonds with ARG218, CYS220, VAL219, and LEU41 (Figure 7).

Followed by the top interaction of ALDH1A1, prednisone formed the van der Waals bonds with ASP122, VAL174, ASN170, LEU428, LEU270, THR245, MET175, and CYS303; conventional hydrogen bonds with TYR297 and GLU269; a carbon-hydrogen bond with SER121 (bond distances in Table 6); pi-sigma bonds with PHE171 and PHE466; and alkyl bonds with VAL460, TRP178, CYS302, and ILE304.

The daunorubicin formed the van der Waals bonds with HIS293, GLY458, ILE304, CYS302, MET175, VAL174, THR129, ALA462, SER461, GLY125, LYS128, and SER121; a carbon-hydrogen bond with VAL460 (bond distances in Table 6); pi-pi stacked with TYR297 and PHE171; and an alkyl bond with TRP178. The doxorubicin formed the van der Waals bonds with LYS128, ALA462, SER461, THR129, VAL174, MET175, THR245, CYS302, ILE304, GLY458, HIS293, and SER121; carbon-hydrogen bonds with VAL460 and GLY125(bond distances in Table 6); pi-pi stacked bonds with PHE171 and TYR297; and an alkyl bond with TRP178 (Figure 6).

In case of CFD, glasdegib formed the van der Waals bonds with SER94, GLU60, ALA61, ASP61, ASP84, GLY62, ALA88, and LEU59; a conventional hydrogen bond with LYS63; carbon-hydrogen bonds with ALA61B and VAL64 (bond distances in Table 6); and pi-alkyl bonds with ALA56, PRO90, and VAL85. The enasidenib formed the van der Waals bonds with LEU104, ASP84, GLN65, GLY62, ALA61B, and ARG87; conventional hydrogen bonds with VAL64, LYS63, VAL85, and LEU86; a carbon-hydrogen bond with PRO90 (bond distances in Table 6); a halogen (fluorine) bond with ALA88; and alkyl bonds with LEU59, ALA61A, and VAL89. Cerubidine formed the van der Waals bonds with LEU86, ARG87, PRO90, ASP61C, ASP61, ALA61, ALA61A, GLY62, VAL64, LYS63, and VAL85; conventional hydrogen bonds with ALA88 and ALA61B (bond distances in Table 6); and a pi-alkyl bond with LEU59 (Figure 7). Our docking studies determined that among the FDA-approved compounds, enasidenib and gilteritinib exhibited the best binding interaction with ALDH1A1 and CFD, respectively; thus, they are the best therapeutic compounds for AML treatment.

### 3.9. Molecular Dynamic Simulation

Molecular dynamics (MD) is used for analyzing the physical movements of atoms and molecules. Among the drug candidates, enasidenib and gilteritinib were chosen for dynamic simulation analysis after a virtual screening. To understand the dynamic activity of the protein-ligand complex in a solvent environment over time, MD simulations were carried out on the YASARA structure tool v. 20.12.24.W.64 (using the AMBER14 force field) with 100 ns.

**Figure 8:**
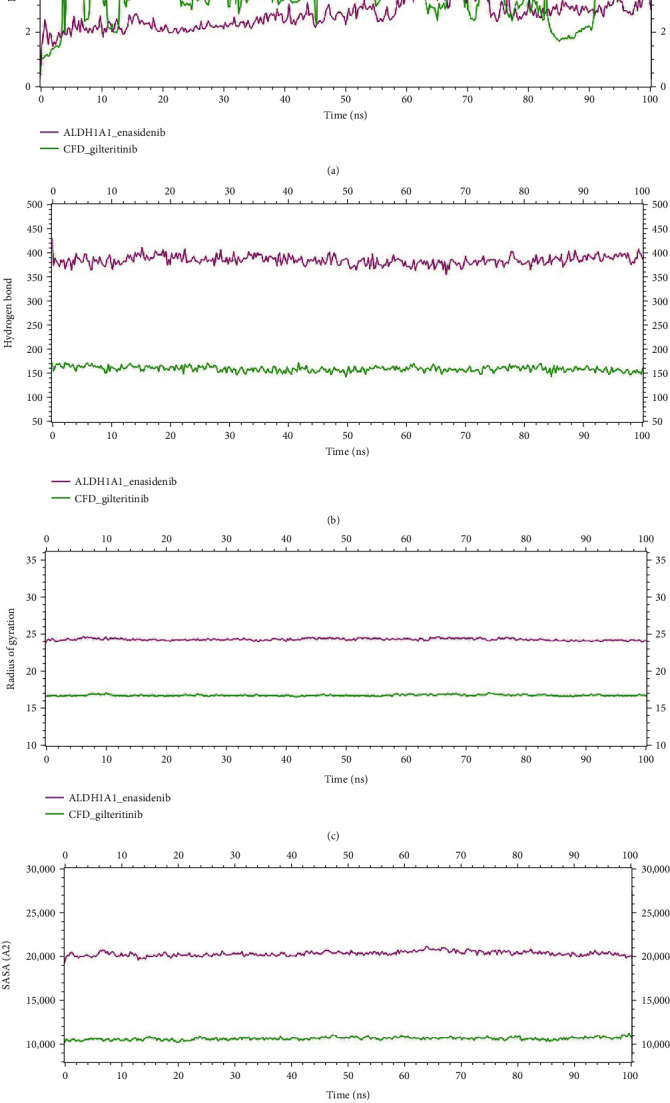
Molecular dynamic simulation of ALDH1A1 and CFD. (a) Root mean square deviation (RMSD), (b) hydrogen bond, (c) radius of gyration, and (d) solvent accessible surface area (SASA) of protein-ligand complexes versus simulation times of 100 ns. Here, complexes: pink: ALDH1A1_enasidenib and green: CFD_gilteritinib.

Figure 8 indicates that the systems were remarkably stable between the moving variation and the initial state of the protein-ligand complexes.  Figure 8(a) represents the RMSD of the ALDH1A1-enasidenib complex and CFD-gilteritinib complex. ALDH1A1_enasidenib complex and CFD_gilteritinib complex show the RMSD around 1.7 Å to 3.4 Å and 1.8 Å to 5.8 Å, respectively. The average RMSD for the ALDH1A1-enasidenib complex and CFD-gilteritinib complex were 1.7 Å, and 4 Å. CFD-gilteritinib complex showed slight fluctuation in 27 ns, 39 ns, and 63 ns and stabilized in the remaining simulation. As can be seen from the plot, the ALDH1A1-enasidenib complex showed a more rigid conformation and achieved stability after 12 ns and remained stable in the rest of the simulation. The number of hydrogen bonds was also used to assess protein stability and folding success. For both simulations, the hydrogen bond analysis revealed an increasing number of bonds with respect to time over the course of the 100 ns ((Figure 8(b)).

Figure 8(c) depicts the radius of gyration for both simulated protein complexes. The radius of gyration is calculated using the center of mass of the protein, which indicates how compact the protein structure is. It will stay constant if the protein is stable; however, it will change over time due to instability. Importantly, both ALDH1A1_enasidenib and CFD_gilteritinib complexes showed no fluctuation in our study, indicating their stability over time.

Additionally, the SASA or solvent-accessible surface areas of the four complexes was evaluated to see if the protein surface or volume had changed. In this study, the total SASA was computed which denotes the biomolecular surface area is accessible to solvent molecules. A higher SASA profile is associated with a longer protein volume, whereas a lower SASA profile is associated with a shorter protein volume. The SASA profile for both complexes had a higher profile and exhibited a stable profile during simulation Figure 8(d).

**Figure 9:**
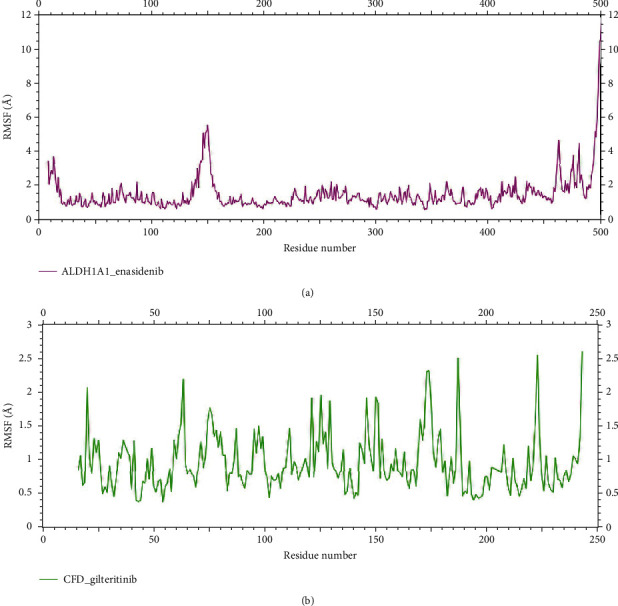
Root means square fluctuation (RMSF) per solute amino acid residues through 100 ns. (a) ALDH1A1_enasidenib complex (pink). (b) CFD_gilteritinib complex (green).

The root mean square fluctuation (RMSF) plot (Figure 9) revealed residual-wise fluctuations, where RMSF/solute residue was calculated using the RMSF of each atom included in the residue. The RMSF for the ALDH1A1_ enasidenib (Figure 9(a)) and CFD_ gilteritinib complexes (Figure 9(b)) were found to be 6 Å and 2.1 Å, respectively.

**Figure 10:**
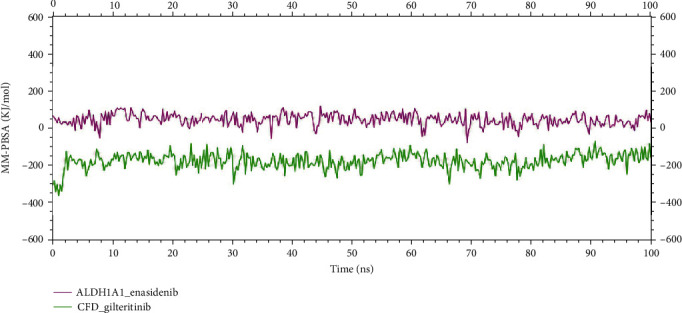
The binding free energy (KJ/mol) of each complex was calculated through 100 ns molecular dynamics simulation based on MM-PBSA, representing the change in binding stability of each complex during simulation. Here, complexes: pink: ALDH1A1_enasidenib and green: CFD_gilteritinib.

Besides, as mentioned previously, the calculation of MM-PBSA binding energy for two complexes was done.  Figure 10 illustrates binding energy with the top-ranked two potential biomarkers, ALDH1A1 and CFD. On average ALDH1A1_enasidenib complex and CFD_gilteritinib complex produced binding energy of 40.588 KJ/mol and -178.766 KJ/mol, respectively. Here, the negative value indicates better binding energy. The 2D interaction has also been analyzed followed by the completion of the 100 ns MD simulation (Figure S. 2).

## 4. Discussion

AML is the most common in adults, which is a grievous, life-threatening, and often remediable hematological malignancy that influences the progenies of myeloid cells and individuals of all ages. Even though the availability of numerous treatments, such as chemotherapy, allogeneic hematopoietic stem cell transplantation (alloHSCT), and receptor-antagonist medications, the 5-year survival of the patient is less than 30% [41]. As a result, research into the biomarkers and precise targets linked to the development of AML could increase diagnostic validity and reduce the financial burden. Integrated bioinformatics analysis has recently become popular for identifying prognostic biomarkers in malignant tumors [42]. Research demonstrated that gene expression differences between normal and malignant tissues might have prognostic significance [43]. Therefore, bioinformatics methods were used to analyze the GSE68925 and GSE183817 datasets from GEO and find biomarkers for the early diagnosis and prognosis of AML. GO and KEGG studies were conducted with the integrated DEGs and revealed the enriched pathway. The PPI network of the integrated DEGs was built using STRING [21]. The PPIs are crucial in establishing and executing intracellular communication and programmed cell death [44]. The method of cytoHubba plugin's MCC topology was used to identify 15 hub genes. Finally, two biomarkers related to AML prognosis were identified by analyzing the survival rate for further analysis. Between these two genes, *CFD* is upregulated, and *ALDH1A1* is downregulated. The GEPIA tool was also used to validate the expression of the two genes. Such data validation confirmed the relation between the selected biomarkers and AML prognosis.

A similar study identified that the scavenger receptor stabilin-1 (*STAB1*) is a prognostic factor of AML that were validated with three other independent CN-AML datasets [45]. In another research, the prognostic indicator, *CALCRL*, was used for determining the chemotherapy schedule and risk of HSCT in patients with AML/ETO+AML [46]. Furthermore, *ERCC3* was considered as a biomarker as its overexpressed elevated *ERCC3* expression in AML patients [47]. Similarly, *HSPA8* high expression was seen in another study in AML, and it was recognized as a possible independent prognostic factor in CN-AML patients [48]. *STAT1*, *BATF*, and *EML4* were identified as independent indicators of pediatric AML [49]. AML patients with aberrant *MMP7* or *MMP15* expression have a substantially poor prognosis, and this finding raises the possibility that *MMP7* and *MMP15* are potential prognostic markers and therapeutic targets for AML [50]. Furthermore, another study identified *POBEC3G* gene as a potential prognostic marker of AML [51].

### 4.1. CFD

The *CFD* gene encodes a serine peptidase protein, a member of the S1, or chymotrypsin family [52]. This protease catalyzes the cleavage of factor B, the rate-limiting step of the alternative pathway of complement activation. This protein also functions as an adipokine, a cell signalling protein secreted by adipocytes, which regulates insulin secretion in mice. Mutations in this gene underlie *CFD* deficiency, which is associated with recurrent bacterial meningitis infection in human patients. Alternative splicing of this gene results in multiple transcript variants, of which at least one variant encodes a preproprotein that is proteolytically processed to generate the mature protease. CFD is a human protein, which is also known as adipsin highly expressed in adipose tissue [53]. Its expression has been associated with AML prognosis, and this imperiled marker may help in better estimation of patient risks [46]. Pediatric AML was caused due to the significant overexpression of *CFD* [54]. It regulates the alternative complement pathway and the production of the complement component C3a, which helps beta cells secrete more insulin [55] that regulates the sugar level in the human body. Increased adipokine production in obesity affects various functions, including appetite and energy balance, insulin sensitivity, blood pressure, immunity, angiogenesis, hemostasis, and lipid metabolism, all of which are also linked to cardiovascular diseases [56]. Moreover, adipsin increases the proliferation of cancer stem-like cells (CSCs), the properties of xenograft (PDX) cells developed in patients with breast cancer [57]. Furthermore, adipsin is a rate-limiting enzyme involved in activating the innate immune system in various malignancies [35–38]. In this study, the hub gene *CFD* showed the highest association with AML and was found to be highly expressed in tumor tissues compared to surrounding normal tissues. Further confirmation of the link between *CFD* and the development of AML may lead to the identification of new targets for AML treatment.

### 4.2. ALDH1A1

Another gene, *ALDH1A1*, responsible for AML belongs to the ALDH superfamily of nineteen different ALDH functional genes [38–40, 58]. Through the NAD(P)^+^-dependent oxidation, the ALDH gene family appears as a varied set of proteins that detoxifies exogenous and endogenous aldehydes [59]. It is likely to play a role in tumor invasion, metastasis, and prognosis and could be a suitable target for predicting gastric cancer prognostic [60]. It also contributes to the detoxification of various regularly used anticancer medications and has a strong activity for the oxidation of aldophosphamide [61].

*ALDH1A1* has recently been linked to the prognosis of several human cancers, including breast cancer, lung cancer, ovarian cancer, and esophageal cancer. [41–44] and has an adverse prognostic effect on colorectal cancer [52, 62]. This biomarker regulates the activation of the AKT signal pathway and interacts with the beta-catenin which could be one of the mechanisms, by which it maintains the characteristics of esophageal squamous cell carcinoma (ESCC) and cancer stem-like cells (CSCs) [63]. ALDH1A1 functions as an isozyme that catalyzes the oxidation of retinaldehyde into retinoic acid followed by the formation of retinol/vitamin A in downstream of retinol dehydrogenases. This is a vital pathway for regulating the amounts of two key chemicals, retinol, and retinoic acid, which can be teratogenic and cytotoxic in case of its excessive production. It has an amino butyraldehyde dehydrogenase activity and is thought to be a part of an alternate pathway for the production of GABA/4-aminobutanoate in the midbrain, possibly contributing to GABAergic synaptic transmission. *ALDH1A1* regulates either RA-driven target genes connected to aggressiveness/stem cell activities or genes with RA response elements (RAREs) in CSCs from human melanoma, making this isozyme a potential therapeutic target in melanoma [64]. According to the GeneCards, no evidence is found in support of the relationship between the *ALDH1A1* and AML. Thus, further investigation is needed to verify the association between *ALDH1A1* and AML and evaluate the gene as a target for AML treatment. Although the precise biological functions of the *CFD* have only been studied by molecular biology approaches [30, 31], this research has conducted a bioinformatics investigation to verify that both the *ALDH1A1* and *CFD* are related to AML. Since this result has been found by the computational analysis of two data sets only, it needs to be validated by techniques of molecular biology, such as RT-PCR and western blot, using clinical samples.

### 4.3. Molecular Docking

Molecular docking has been performed to find out the most effective drugs for AML from the list approved by the FDA. The first prediction of molecular recognition between ligand and target is heavily influenced by molecular docking. Molecular docking reveals the interacting amino acid residues, docking energy analysis, hydrogen bonding, and analysis of amino acid residues of the active sites and potential binding sites, which were used to decipher drug-target interactions [65]. Various types of atomic/molecular attractions, such as van der Waals forces, hydrophobic bonds, and hydrogen bonds, contribute to the accumulated binding strength of the interacting complexes [57, 66]. At the prominent and active binding sites, hydrogen bonding is influenced by the composition and 3D alignment of interacting amino acid residues [67]. Based on the binding pattern, the top-scored protein-ligand interactions for each of the biomarkers were predicted and selected as the best-matched agonist.

### 4.4. Molecular Dynamic Simulation

The best compound was selected by MD simulation and free energy calculation on the top-scoring compounds (Tables 4 and 5). Due to the significant energy dissipation for each conformation, this technique takes a longer time to determine the best-docked conformer. This approach has the advantage of being more compatible with accepting ligand flexibility. Furthermore, it has fidelity in evaluating the inhibition of the target proteins. According to the plot of RMSD, hydrogen bond, Rg, SASA, RMSF, and MM-PBSA simulation, the two complexes indicated insignificant fluctuation in 100 ns resulting in good binding stability. According to a previous *in silico* research [68], the ellipticine reactive metabolites 13-hydroxyellipticine and 12-hydroxyellipticine are likely to be effective drugs for treating breast cancers with strong *ALDH1A1* activity. The research showed that the protein backbone RMSD (1.55 Å) revealed that the structure was stabilized after 3,000 ps of simulation. Another study [69] screened selective ALDH1A1 inhibitors and identified the top four hits (ALDH-D1, ALDH-D3, ALDH-D4, and ALDH-D5) for dynamic simulation. The RMSD plots of all docked complexes were plotted for 30 ns, and it was observed that each complex is in a steady state and showed minimum RMSD fluctuations during the entire simulation period. A similar study [70] investigated 14 missense SNPs by dynamic simulation and revealed the impact of missense SNPs on the metabolic resistance to cyclophosphamide caused by ALDH1A1-mediated mutations. In this case, the RMSD plot of the wild type demonstrates its stability with little fluctuations, i.e., 1 Å (1.9–2.9 Å). According to our study, the ALDH1A1-enasidenib complex and the CFD-gilteritinib complex had average RMSDs of 1.7 and 4, respectively.

## 5. Conclusion

In summary, our investigation found 238 DEGs by integrating the two GEO datasets of AML. The PPI networks and survival analysis identified two important genes, *ALDH1A1* and *CFD*, that are strongly associated with the progression of tumors and cancer. This suggests that the two biomarkers are likely to serve as prognostic indicators and therapeutic targets for AML. They had great docking energy with the FDA-approved AML drugs, gilteritinib and enasidenib, respectively. Moreover, the MD simulation validated the stability of their binding between active pockets of the proteins and compounds. This study also ensured the stages and clinical diagnosis of AML and, finally, provided the specific medication for a specific patient. The *in silico* results presented in this article need to be validated by laboratory research, which would be our future target. That is why, the authors are interested to confirm the function of these screened genes and pathways in the development of AML through future study by laboratory research (*in vitro* and *in vivo*).

## Figures and Tables

**Table 1:** Details of the AML data in GEO.

Accession no.	Platform	Control	Clinical case	DEGs counts
GSE68925	GPL11154	03	04	1965
GSE183817	GPL20301	03	04	1449

**Table 2:** List of the integrated DEGs in AML.

Upregulated	*ADAMTS2*, *RFX8*, *MMP19*, *CLEC5A*, *PCAT18*, *PTPN14*, *CCDC189*, *PIWIL4*, *PLPPR3*, *SCUBE1*, *PTX3*, *HOMER3*, *PPP1R27*, *GGT5*, *SERPINE1*, *KCNN4*, *LOC105377267*, *HHIP*, *RHOB*, *ARL4A*, *PRG2*, *MICALL2*, *SLC1A3*, N*CF4*, *HLA-DQB1*, *IGSF10*, *PLXNB1*, *CXCL2*, *MROH7*, *CCNA1*, *LAIR1*, *CFD*, *HOXB6*, *COL23A1*, *RNASE2*, *AZU1*, *FAM227A*

Downregulated	*SLC25A23*, *SYDE2*, *ANKH*, *MAGEE1*, *LOC107985075*, *LDOC1*, *IFFO2*, *TAMM41*, *BCAS4*, *LGR6*, *KCNA3*, *RPP21*, *SFXN1*, *UBASH3A*, *ZDBF2*, *SCML4*, *TAPBP*, *ALB*, *TTC39B*, *AKT3*, *AKAP12*, *ALDH1A1*, *ABLIM1*, *CYP4F22*, *PHGDH*, *KLRG1*, *SKAP1*, *LEF1*, *C3orf14*, *NAP1L3*, *APBB1*, *DTX3*, *ADGRG1*, *GATA3*, *NUDT11*, *TUBB*, *RHOF*, *CPED1*, *NFATC2*, *HLA-DPB1*, *FAT4*, *TC2N*, *HOPX*, *TCEAL2*, *PRSS23*, *TRPC1*, *MAGI2-AS3*, *LZTS3*, *GRAP2*, *ICOS*, *HOOK1, FAM169A*, *CYFIP2*, *PBX1*, *HLA-DPA1*, *SALL2*, *BEX5*

**Table:** (a) Significant GO analysis of the integrated upregulated DEGs

Category	Term	Count	*p* value	Genes
Biological process	Inflammatory response	5	0.004964045	*GGT5*, *SCUBE1*, *PTX3*, *AZU1*, *CXCL2*
	Immune response	4	0.041879060	*NCF4*, *PRG2*, *CXCL2*, *HLA-DQB1*

Cellular component	Extracellular region	12	5.56E-05	*CFD*, *IGSF10*, *ADAMTS2*, *HHIP*, *SERPINE1*, *PRG2*, *MMP19*, *PLXNB1*, *PTX3*, *AZU1*, *RNASE2*, *CXCL2*
	Extracellular space	7	0.027764289	*CFD*, *SCUBE1*, *SERPINE1*, *MROH7*, *PTX3*, *AZU1*, *CXCL2*
	Plasma membrane	13	0.040982851	*GGT5*, *COL23A1*, *SERPINE1*, *SLC1A3*, *RHOB*, *ARL4A*, *CLEC5A*, *MICALL2*, *HOMER3*, *PLXNB1*, *KCNN4*, *LAIR1*, *HLA-DQB1*
	Endosome membrane	3	0.041660003	*NCF4*, *RHOB*, *HLA-DQB1*

**Table:** (b) Significant GO analysis of the integrated downregulated DEGs

Category	Term	Count	*p* value	Genes
Biological process	T cell receptor signalling pathway	5	6.52*E* − 04	*GRAP2*, *HLA-DPB1*, *GATA3*, *SKAP1*, *HLA-DPA1*
	T cell costimulation	4	0.001188945	*GRAP2*, *HLA-DPB1*, *ICOS*, *HLA-DPA1*
	Positive regulation of signal transduction	3	0.011624363	*GRAP2*, *GATA3*, *SKAP1*
	Regulation of gene expression	3	0.029442346	*PHGDH*, *TC2N*, *TAPBP*
	Embryonic hemopoiesis	2	0.042044862	*GATA3*, *PBX1*
	Antigen processing and presentation of peptide or polysaccharide antigen via MHC class II	2	0.044614484	*HLA-DPB1*, *HLA-DPA1*
	Positive regulation of T cell activation	2	0.047177366	*HLA-DPB1*, *HLA-DPA1*
	Transcription from RNA polymerase II promoter	5	0.047916492	*SALL2*, *LEF1*, *NFATC2*, *GATA3*, *PBX1*
	Positive regulation of transcription, DNA templated	5	0.048484768	*LEF1*, *NFATC2*, *GATA3*, *APBB1*, *SKAP1*

Cellular component	An integral component of the lumenal side of the endoplasmic reticulum membrane	3	0.002970387	*HLA-DPB1*, *HLA-DPA1*, *TAPBP*
	Trans-Golgi network membrane	3	0.022619857	*HLA-DPB1*, *LGR6*, *HLA-DPA1*

Molecular function	Peptide antigen binding	3	0.002513237	*HLA-DPB1*, *HLA-DPA1*, *TAPBP*
	Transcription factor binding	5	0.006798913	*LEF1*, *NFATC2*, *GATA3*, *APBB1*, *PBX1*
	Transcriptional activator activity, RNA polymerase II core promoter proximal region sequence-specific binding	4	0.024880383	*LEF1*, *NFATC2*, *GATA3*, *PBX1*
	MHC class II receptor activity	2	0.039264179	*HLA-DPB1*, *HLA-DPA1*
	MHC class I protein binding	2	0.049477439	*TUBB*, *TAPBP*

**Table:** (c) Significant pathway analysis of the integrated upregulated DEGs

Category	Term	Count	*p* value	Genes
KEGG_pathway	T cell receptor signalling pathway	4	0.00207572	*GRAP2*, *AKT3*, *NFATC2*, *ICOS*
	Intestinal immune network for IgA production	3	0.006520509	*HLA-DPB1*, *ICOS*, *HLA-DPA1*
	Antigen processing and presentation		0.016438175	*HLA-DPB1*, *HLA-DPA1*, *TAPBP*
	HTLV-I infection	4	0.026924735	*AKT3*, *HLA-DPB1*, *NFATC2*, *HLA-DPA1*
	Toxoplasmosis	3	0.032814588	*AKT3*, *HLA-DPB1*, *HLA-DPA1*
	Epstein-Barr's virus infection	3	0.039663289	*AKT3*, *HLA-DPB1*, *HLA-DPA1*

**Table 4:** Docking score of top four compounds with ALDH1A1.

Ligand	Binding affinity (kcal/Mol)	Amino acid residues of the active site
Enasidenib	-10.8	GLY125, VAL174, THR129, SER121, VAL460, TYR297, TRP178, PHE171, PHE466, MET175ILE304, HIS293, CYS302, CYS303, GLY294, TYR497, GLY498
Prednisone	-10.7	TYR297, SER121, ASP122, ASN170, LEU428, LEU270, ILE304, CYS302, CYS303, VAL460, VAL174, PHE171, PHE466, MET175, TRP178, THR245, GLU269
Daunorubicin	-10.5	TYR297, SER121, ILE304, CYS302, VAL460, VAL174, PHE171, MET175, TRP178, THR129, ALA462, GLY458, HIS293, SER461, LYS128
Doxorubicin	-10.1	PHE171, CYS302, ILE304, TYR297, GLY458, HIS293, SER121, VAL460, LYS128, GLY125, ALA462, TRP178, SER461, THR129, VAL174, MET175, THR245

**Table 5:** Docking score of the top four compounds with CFD.

Ligand	Binding affinity (kcal/Mol)	Amino acid residues of the active site
Gilteritinib	-8.3	CYS58, LEU41, CYS42, GLY193, SER195, CYS191, GLY216, ARG218, CYS220, ILE143, VAL219, SER217, SER215, LYS192, HIS57, GLU60
Glasdegib	-7.7	SER94, GLU60, ALA61, ASP61, ALA61B, VAL85, VAL64, LYS63, ASP84, GLY62, ALA88, LEU59, PRO90, ALA56
Enasidenib	-7.3	PRO90, LEU59, ALA61A, ALA61B, VAL89, ALA88, LEU86, ARG87, VAL85, GLY62, LYS63, GLN65, VAL64, ASP84, LEU104
Cerubidine	-7.2	LEU86, ARG87, ALA88, LEU59, PRO90, ASP61C, ASP61, ALA61, ALA61B, ALA61A, GLY62, VAL64, LYS63, VAL85

**Table 6:** Interaction profile of the selected complexes with hydrogen bond distance.

Name of the targets	Name of the ligands	Interacting amino acids	Bond distance in Å	Type of interaction
ALDH1A1	Enasidenib	GLY294	3.47	Carbon hydrogen bond
		GLY458	3.8	
	Prednisone	GLU269	3.38	Conventional hydrogen bonds
		TYR297	6.28	
		SER121	3.39	Carbon hydrogen bond
	Daunorubicin	VAL460	4.88	Carbon hydrogen bond
	Doxorubicin	GLY125	3.22	Carbon hydrogen bond
		VAL460	4.87	

CFD	Gilteritinib	LEU41	5	Conventional hydrogen bonds
		GLY193	3.27	
		SER215	4.28	
		GLY216	5.52	Carbon hydrogen bond
		VAL219	4.6	
	Glasdegib	LYS63	4.1	Conventional hydrogen bonds
		ALA61B	5.02	Carbon hydrogen bond
		VAL64	4.13	
	Enasidenib	LYS63	3.32	Conventional hydrogen bonds
		VAL64	3.85	
		VAL85	4.29	
		LEU86	4.72	
		PRO90	4.79	Carbon hydrogen bond
	Cerubidine	ALA61B	4.13	Conventional hydrogen bonds
		ALA88	3.33	

## Data Availability

The data used to support the findings of this study are included within the article.

## Supplementary Materials

Supplementary Materials Figure S. 1: survival analysis of 13 nonsignificant hub genes in AML (p >0.05). (a) *PHGDH*, (b) *SERPINE* 1, (c) *ALB*, (d) *GATA3*, (e) *GRAP2*, (f) *HLA*-*DPA1*, (g) *HLA*-*DPB1*, (h) *HLA*-*DQB1*, (i) *ICOS*, (j) *KCNN4*, (k) *LEF1*, (l) *NFATC2*, and (m) *PBX1*. Figure S. 2: 2D interaction diagram after completing the 100 ns MD simulation. (a) ALDH1A1_ Enasidenib complex and (b) CFD_ Gilteritinib complex.
