# Supplementary material for: Exploring Prognostic Biomarkers of Acute Myeloid Leukemia to Determine Its Most Effective Drugs from the FDA-Approved List through Molecular Docking and Dynamic Simulation
Source: Biomed Res Int. 2023 Jun 15;2023:1946703. doi: 10.1155/2023/1946703 (PMC10287530; doi:10.1155/2023/1946703)
Supplement: Supplementary Materials — Figure S. 1: survival analysis of 13 nonsignificant hub genes in AML (p >0.05). (a) PHGDH, (b) SERPINE 1, (c) ALB, (d) GATA3, (e) GRAP2, (f) HLA-DPA1, (g) HLA-DPB1, (h) HLA-DQB1, (i) ICOS, (j) KCNN4, (k) LEF1, (l) NFATC2, and (m) PBX1. Figure S. 2: 2D interaction diagram after completing the 100 ns MD simulation. (a) ALDH1A1_ Enasidenib complex and (b) CFD_ Gilteritinib complex. [file 1946703.f1.docx]

**Supplementary**
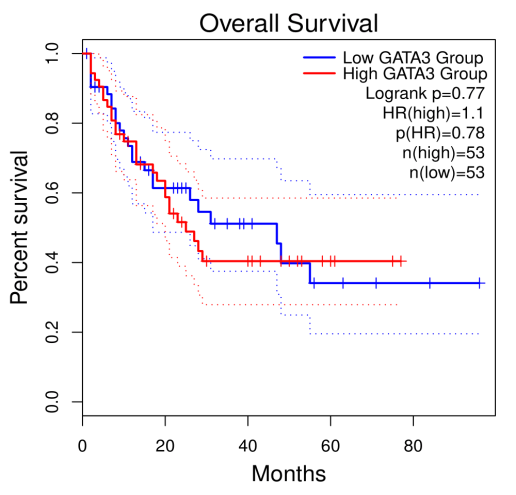

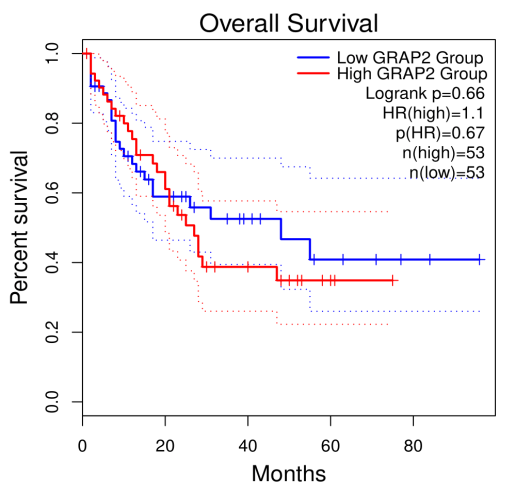
**Figures**


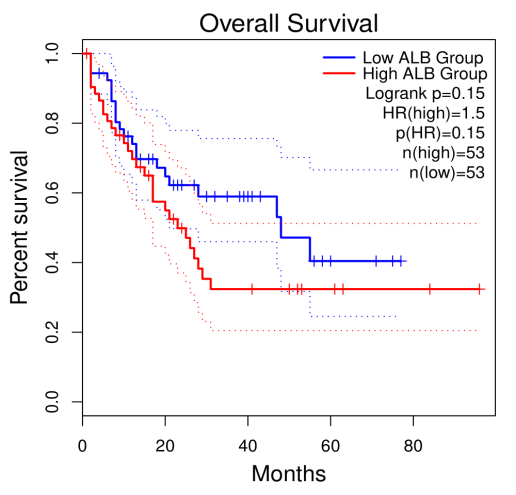

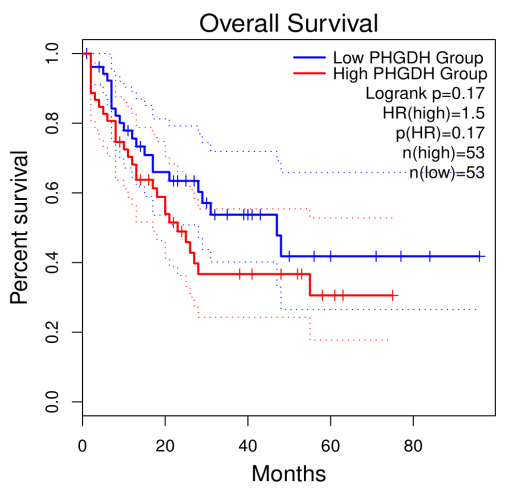

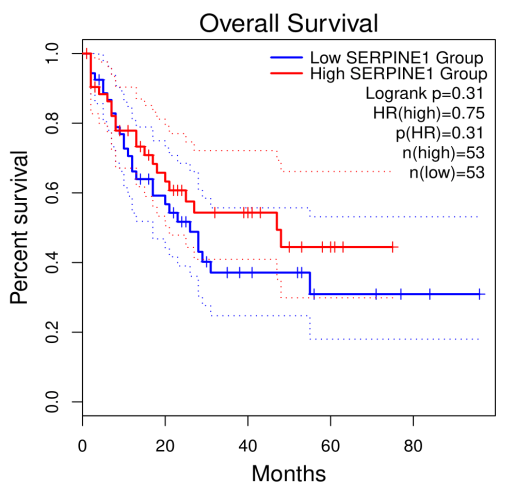


(c)

(b)

(a)


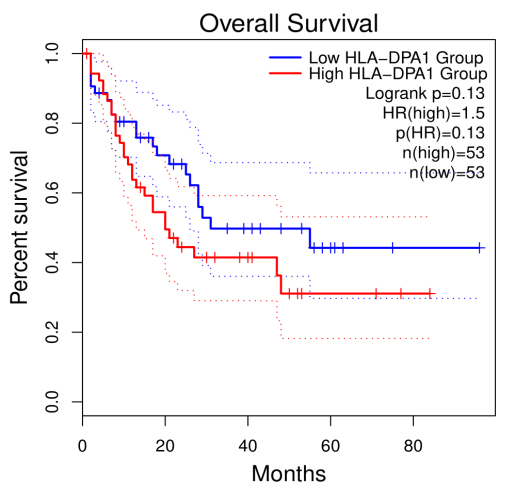


(d)

(e)

(f)


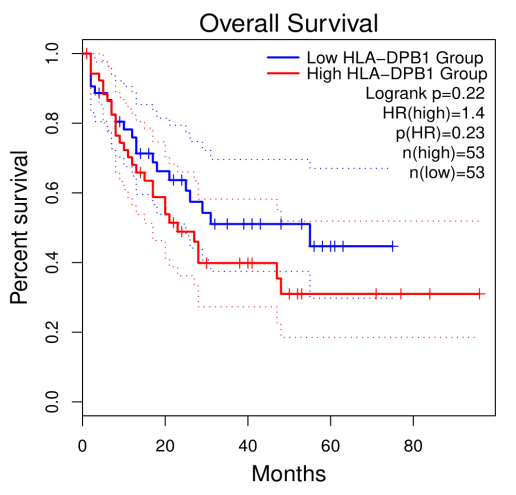

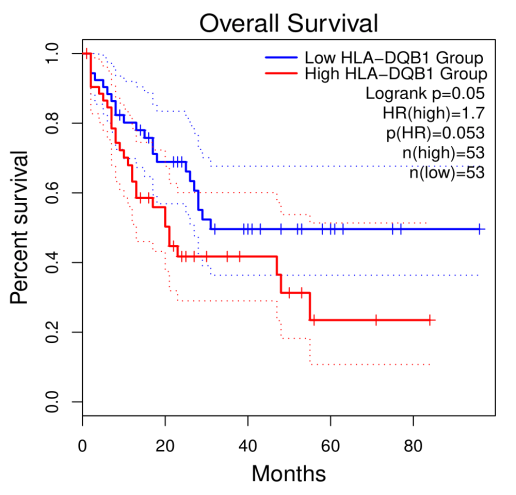

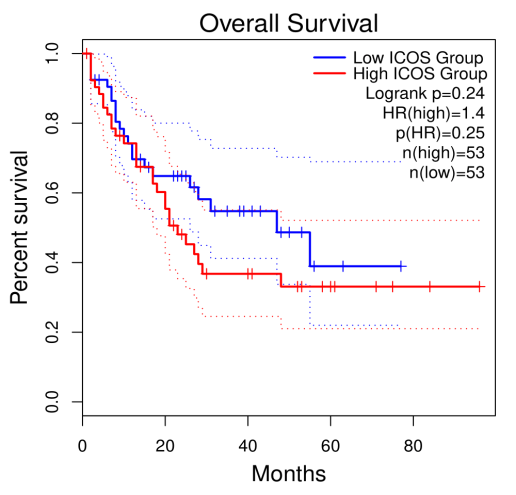


(i)

(h)

(g)


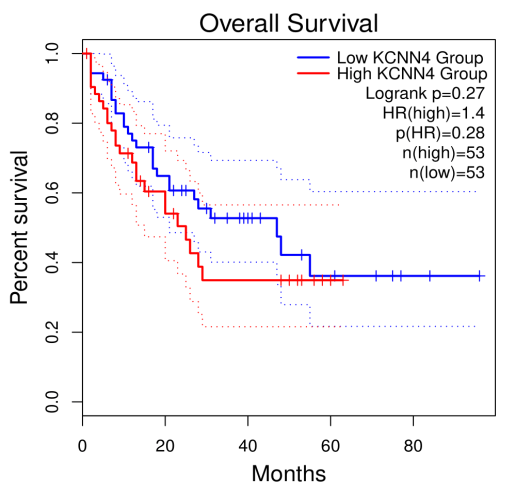

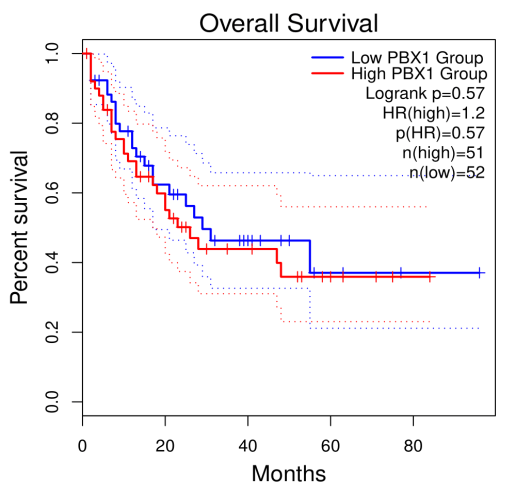

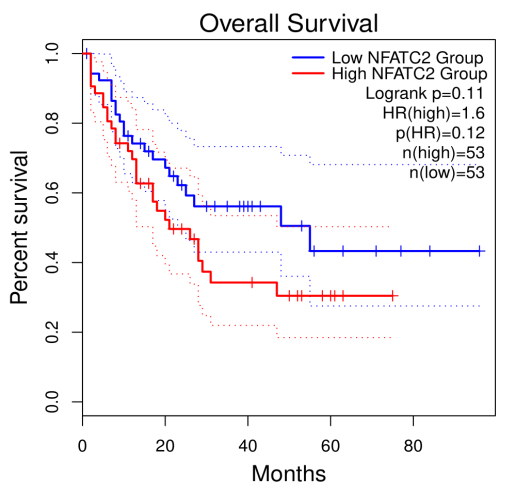

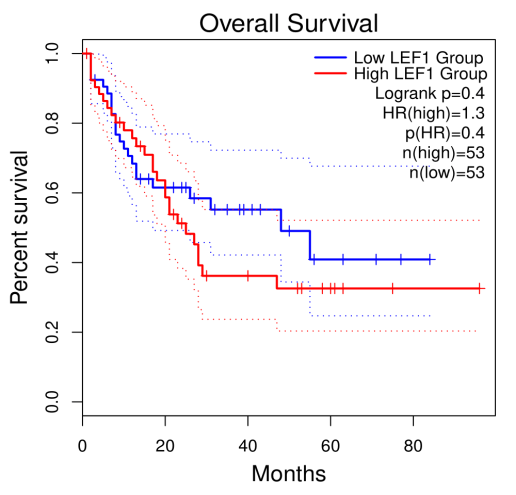


(m)

(l)

(k)

(j)

**Figure S. 1:** Survival analysis of 13 non-significant hub genes in AML (p>0.05). (a) *PHGDH*, (b) *SERPINE* 1, (c) *ALB*, (d) *GATA3*, (e) *GRAP2*, (f) *HLA*-*DPA1*, (g) *HLA*-*DPB1*, (h) *HLA*-*DQB1*, (i) *ICOS*, (j) *KCNN4*, (k) *LEF1*, (l) *NFATC2*, (m) *PBX1*.


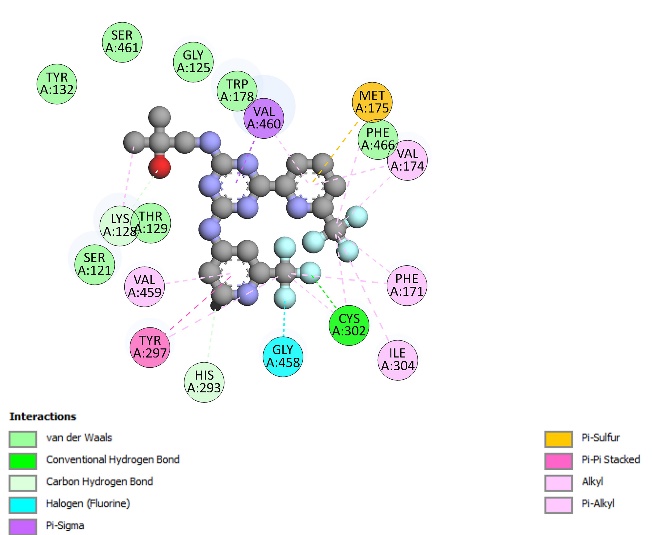

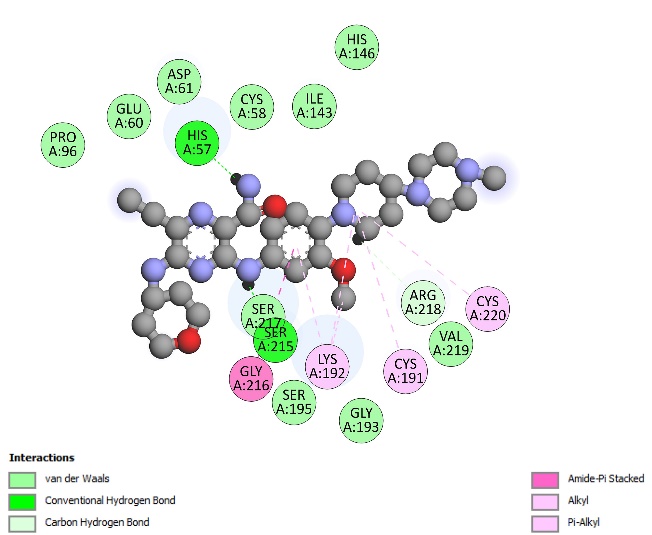


**Figure S. 2:** 2D interaction diagram after completing the 100 ns MD simulation. (a) ALDH1A1_ Enasidenib complex, (b) CFD_ Gilteritinib complex

(a)

(b)
